# Assessing cognitive biases induced by acute formalin or hotplate treatment: an animal study using affective bias test

**DOI:** 10.3389/fnbeh.2024.1332760

**Published:** 2024-01-25

**Authors:** Yu-Han Zhang, Jie-Xuan Lin, Ning Wang, Jin-Yan Wang, Fei Luo

**Affiliations:** ^1^CAS Key Laboratory of Mental Health, Institute of Psychology, Chinese Academy of Sciences, Beijing, China; ^2^Department of Psychology, University of Chinese Academy of Sciences, Beijing, China

**Keywords:** pain, cognitive bias, affective bias test, hotplate, formalin

## Abstract

Pain, a universal and burdensome condition, influences numerous individuals worldwide. It encompasses sensory, emotional, and cognitive facets, with recent research placing a heightened emphasis on comprehending pain’s impact on emotion and cognition. Cognitive bias, which encompasses attentional bias, interpretation bias, and memory bias, signifies the presence of cognitive distortions influenced by emotional factors. It has gained significant prominence in pain-related research. Human studies have shown that individuals experiencing pain exhibit cognitive bias. Similarly, animal studies have demonstrated cognitive bias in pain-induced states across various species and disease models. In this study, we aimed to investigate the memory bias displayed by rats experiencing acute pain, using the affective bias test (ABT) as a tool and administering either hotplate or formalin to induce acute pain. Our data showed that rats demonstrated a significant preference for the control treatment-related substrate over the substrate associated with formalin treatment (*p* < 0.001), an indication of the prominent memory bias stimulated by acute formalin injections. However, when exposed to substrates related to hotplate treatment and control treatment, the acute pain induced by the hotplate treatment failed to generate a statistically significant choice bias in rats (*p* = 0.674). Our study demonstrates that the negative emotions associated with acute pain can be reflected by memory bias in ABT, at least for formalin-induced acute pain. This finding will augment our comprehension of the emotional and cognitive aspects of acute pain.

## Background

Pain is a multi-dimensional experience that includes not only sensation-discrimination components but also emotion-motivation and cognitive-evaluation components (Melzack and Wall, 1965). In recent years, there has been a growing focus on understanding the complex involvement of emotion, motivation, and cognitive assessment in the experience of pain (Bushnell et al., 2013). A critical psychological phenomenon that has garnered significant interest is placebo analgesia. Research has well-documented that the expectation of pain relief plays a key role in modulating placebo analgesic effects (Benedetti et al., 2005). Notably, even clinical analgesic doses can be hindered by negative treatment outcome expectations, leading to a reversal of analgesia (Bingel et al., 2011). These findings underscore the need to consider cognitive and emotional factors when assessing pain management interventions. Empirical research has established a bidirectional relationship between pain, cognition, and emotions (Peters, 2015; Malfliet et al., 2017). Prolonged pain can contribute to mental disorders, while mental disorders are associated with increased pain severity (Michaelides and Zis, 2019). Understanding the emotional and cognitive aspects of pain is crucial for effective pain management.

Cognitive biases refer to the tendency of the brain to have a certain emotional valence when processing information, consequently influencing various cognitive processes including attention, decision-making, and memory (Everaert et al., 2012). In the context of pain, there are three primary types of cognitive biases: attentional bias, judgment bias, and memory bias (Todd et al., 2022). Attentional bias in pain involves prioritizing pain-related information, likely as a coping strategy (Schoth et al., 2012; Mennen et al., 2019). Judgment bias leads to interpreting ambiguous cues in a pain-related or disease-related manner for chronic pain patients (Schoth and Liossi, 2016). Memory bias results in recalling life events during painful episodes with a more negative valence (Meyer et al., 2015). Cognitive biases can impact pain prognosis (Apkarian et al., 2011). Individuals with more pain-related biases often report more intense pain (Heathcote et al., 2016). Excessive interpretation of ambiguous pain information as threatening (pain interpretation bias) can drive fear of pain, pain catastrophizing, and subsequent attempts to avoid pain, contributing to the maintenance of pain and disability (Vlaeyen and Linton, 2000; Todd et al., 2023). Therefore, understanding these cognitive biases is vital for a comprehensive grasp of pain mechanisms and effective pain management.

Research on cognitive biases in animals has advanced, paralleling human studies and leading to tools for assessing non-human species (Robinson, 2018). Harding et al. demonstrated for the first time in 2004 that rats in negative affective states exhibit negative explanatory biases due to reduced expectations of rewards when faced with ambiguous cues (Harding et al., 2004). Till now, numerous animal studies have explored cognitive biases for assessing affective changes in animals (Zhang et al., 2022). However, the investigation of pain-induced cognitive biases in animal models is relatively new, and recent studies have shown promising developments. For instance, disbudding in dairy calves has been linked to post-operative pain, resulting in negative interpretation biases (Neave et al., 2013). In another study, rats with intestinal mucosal inflammation and pain showed reduced optimistic decision-making in judgment bias tasks (George et al., 2018). Tajerian et al. (2019) observed that injured mice display a preference for recalling distressing memories, which in turn contributes to the perpetuation of the vicious cycle involving chronic pain and negative emotions. The investigation of chronic neuropathic pain revealed that rats subjected to saphenous nerve ligation displayed a notable preference for substrates associated with rewards following the administration of gabapentin, suggesting a positive choice bias (Phelps et al., 2021). Extensive research supports the presence of cognitive biases induced by pain across various species. Consequently, exploring the cognitive biases prompted by pain in animals holds significance, as it can substantially enhance our comprehension of similar biases observed in the human experience of pain.

The affective bias test (ABT) is a cognitive bias test developed by Robinson’s team in 2013 to measure memory biases in animals under different affective states (Stuart et al., 2013). In the ABT paradigm, rats learn to associate specific substrates with food rewards under varying affective states, and during the testing stage they choose between these substrates that were previously linked with rewards; given that the absolute value of the rewards remains constant during the substrate pairing learning stage, any observable choice bias during the testing stage is attributed to alterations in the affective state brought on by the treatment (Stuart et al., 2013). Previous studies primarily focused on the depressive affective state of rats using the ABT, demonstrating its efficacy in measuring memory biases that arise from negative affective states in animals (Stuart et al., 2013; Hinchcliffe et al., 2017; Stuart et al., 2017). Considering that acute pain can induce a negative affective state (Wiech and Tracey, 2009), this study employs the ABT to investigate whether acute pain results in a negative affective bias in rats. We hypothesize that acute pain will elicit a choice bias in rats based on reward value. This experiment advances our comprehension of the impact of acute pain on cognitive biases, offering valuable insights into the emotional and cognitive dimensions of pain perception.

## Methods

### Animals

Twelve male Sprague Dawley rats (weighing 175–185 g on arrival, purchased from Charles River, Beijing, China) were used in this study. The rats were individually housed in standard cages. The housing environment was rigorously maintained at a controlled temperature of 22°C and approximately 65% humidity, following a 12/12-h light/dark cycle with lights on at 8:00 p.m. Before conducting experiments, rats acclimated for at least a week to handling and reward pellets, with daily interactions and access to a daily food supply including 10 sucrose pellets. On days when experiments were conducted, water was readily available to the rats, except during the execution of the behavioral training and testing. Since the rats had the chance to earn sucrose pellets as part of the task, their food intake was carefully regulated. Their weights ranged from 200 g to 300 g during the experimental phase, and they were allocated a daily food allowance ranging from 12 g to 15 g. Measures were taken to minimize discomfort or pain. All rats were alive upon completion of this research and were used in preliminary experiments for another study. All the experimental procedures were approved by the Institutional Animal Care and Use Committee of the Chinese Academy of Sciences, ensuring the welfare and ethical treatment of the animals.

### Apparatus

The animals were tested in a perspex arena with opaque sides (40 cm × 40 cm × 40 cm). Digging substrates, such as hard paper scraps, cottonwood fiber, and timothy grass, were placed in bowls (Ø 10 cm) and presented in a pseudo-random order in the left or right position within the arena (more details for digging substrates see Supplementary Table S1). A video recorder was placed to record the rats’ behaviors.

### Affective bias test (ABT)

#### Training stage

The entire ABT experiment was executed following the methodology outlined by Stuart et al., including the training stage and the formal experimental stage (Stuart et al., 2013). During the initial training phase, on the first day, two empty bowls were presented to each rat, and five sucrose pellets (45 mg sucrose tablets, Bio-serv, US, #F0023) were placed in each bowl. The rats were given 10 minutes to explore the bowls or until they had consumed all of the pellets. In the following days, corncobs were introduced into the bowls, and the amount of corncobs placed in the bowls gradually increased from 1 cm to 2.5 cm. Inside each of the two corncobs-filled bowls, three sucrose pellets were hidden, and an additional three pellets were placed on top of the corncobs to encourage digging behavior. Each rat was introduced individually into the testing arena and given 10 minutes or until they located and consumed all the pellets within the bowls. When a rat successfully located the pellets in both bowls for six consecutive trials during the initial training, they advanced to the discrimination training phase.

In the discrimination training phase, an opaque baffle was introduced. Before the start of each trial, the rat was positioned behind the baffle. As soon as the trial commenced, the baffle was removed, allowing the rat to move toward and explore the two bowls. One of these bowls contained a substrate concealing a sucrose tablet, while the other bowl contained a substrate without any concealed rewards. The rat was tasked with a choice between the two bowls, and once it began digging in one of the bowls, the other bowl was promptly taken away. To reduce any dependence on olfaction for selection, a reward pellet was crushed and evenly distributed within both bowls of substrates. Digging in the substrate associated with the reward was documented as a correct trial, whereas digging in the substrate without a reward was documented as an incorrect trial. If the rat failed to approach and investigate a bowl within 60 s, the trial was categorized as an “omission.” The session continued until the rat reached a criterion of six consecutive correct trials. All 12 rats completed the training phase and entered the formal experimental stage.

### Formal experimental stage

**Figure 1:**
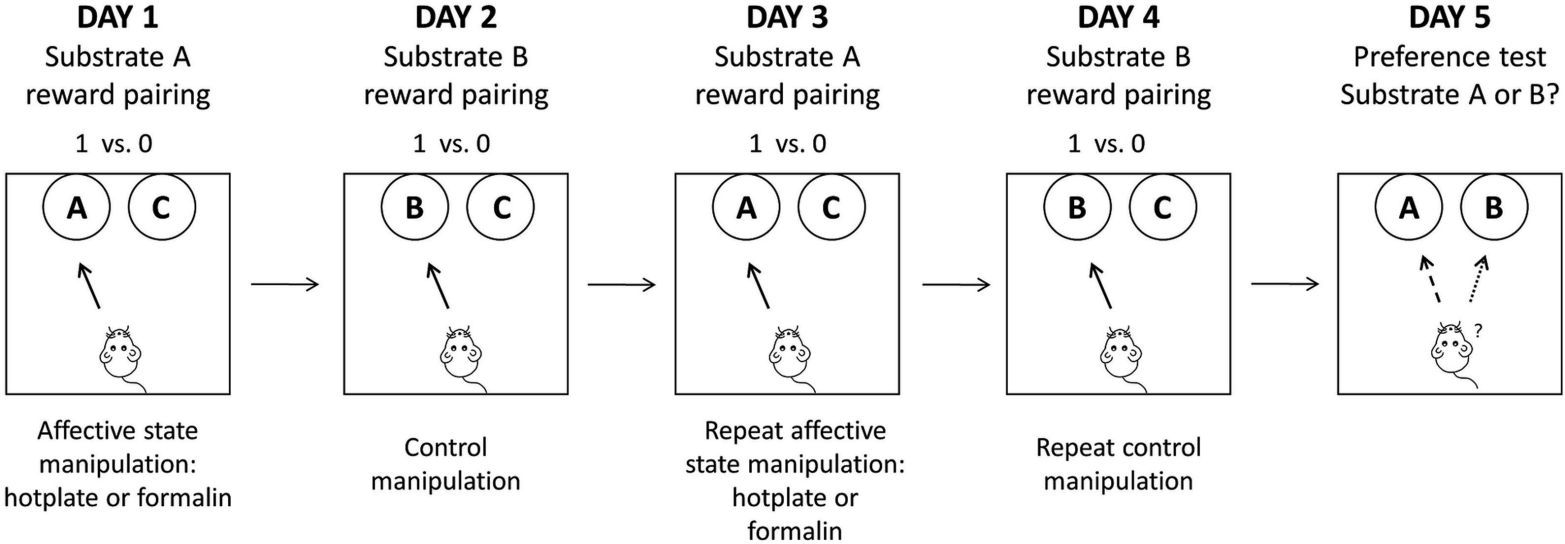
A schematic representation of the formal experimental stage. Rats were exposed to four independent substrate-reward paired learning sessions, each paired with different affective state manipulations. On the fifth day, a preference test was conducted to evaluate the choice bias of rats. Adapted from Stuart et al. (2013).

The formal experiment stage consisted of 4 days of reward-pairing learning followed by a fifth-day preference test. On the reward-pairing learning days, after the removal of the baffle, the rat was granted free access to move and explore two bowls, each containing distinct digging substrates. One reward substrate was associated with acute pain treatment on Day 1 and Day 3 (or Day 2 and Day 4 for counterbalance), while the other reward substrate was associated with the control treatment on Day 2 and Day 4. During each trial, the latency to initiate digging, defined as the time it took for the rat to commence digging behavior in one of the bowls, was precisely recorded. In addition, the total number of trials completed by each rat, along with the aggregate count of trials categorized as ‘omission’, were documented. The session concluded when the rat reached a criterion of six consecutive correct trials within 60 s per trial. The assessment of affective biases was carried out during the preference test on Day 5, during which the two substrates that had previously been associated with rewards (referred to as “substrate A” and “substrate B”) were simultaneously presented across 30 trials, with each substrate has a one-third probability of reward. Refer to Figure 1 for a schematic representation of the formal experimental stage.

### Paradigm verification: effects of the absolute value of the reinforcer (2 pellets vs. 1 pellet)

To examine whether doubling the absolute reward value of one of the substrates could elicit a choice bias between the substrates, an experimental design was employed here wherein one substrate was associated with two reward pellets, while the other substrate was associated with a single reward pellet. On Day 5, during the preference testing stage, both substrates were reinforced with a single sugar pellet, employing a random reinforcement protocol.

### Acute pain treatments

#### Hotplate-induced acute pain

Using the hotplate to induce acute heat pain, the rat was put on the surface of a metal plate with a surface temperature of 52.5°C. The threshold was defined as the latency of the rat licking or lifting the hind paw, or jumping. 30 s is the maximum cut-off time to avoid possible tissue damage (Imanaka et al., 2008). After the rat first licked or lifted the hind paw or jumped, the rat was immediately taken out and placed in the ABT experimental arena to carry out the ABT reward-pairing procedure. One reward substrate was associated with the acute pain pre-treatment, whereas the other reward substrate was associated with the control treatment, involving placing the rats on a turned-off hotplate for 15 s.

#### Formalin-induced acute pain

Formalin was used to induce acute inflammatory pain. 50 μL of a 1% formalin solution was injected subcutaneously into the surface of one hind paw (Liu et al., 2019). The rat was then immediately placed in the ABT experimental arena and carried out the ABT reward-pairing procedure. The control treatment entailed the administration of a 50 μL saline injection to the same hind paw. On the subsequent reward-pairing learning days, the injection was administered to the alternate hind paw. Pain-related behaviors were meticulously quantified as the total duration during which the rat exhibited licking or lifting the hind paw that had received the injection, with observations taken at 5-min intervals over 60 min.

### Analysis

The sample size employed in this study was based on previous investigations (Hinchcliffe et al., 2017, 2020), and further substantiated through a post-hoc power analysis (observed power for all main analyses >0.9). The Prism (version 8; GraphPad Software Inc.) and SPSS (version 25; IBM Corporation, Armonk, NY, USA) software packages were used for graph generation and statistical analyses. % Choice bias = the number of choices made for the treatment-paired substrate / the total number of trials × 100–50% (Stuart et al., 2013). For each treatment, a one-sample *t*-test was used to compare the theoretical mean of 0% choice bias. Analysis of “trials to criterion,” “choice latencies” and “omissions” involved paired *t*-tests comparing the days with affective state manipulation to the control manipulation days. As for the formalin treatment test, the pain-related behavior data were averaged for the first and second injections and were assessed using two-way repeated measures ANOVA followed by Bonferroni’s multiple comparisons test.

## Results

### Evaluation of experiment design validity: 2 pellets vs. 1 pellet test

**Figure 2:**
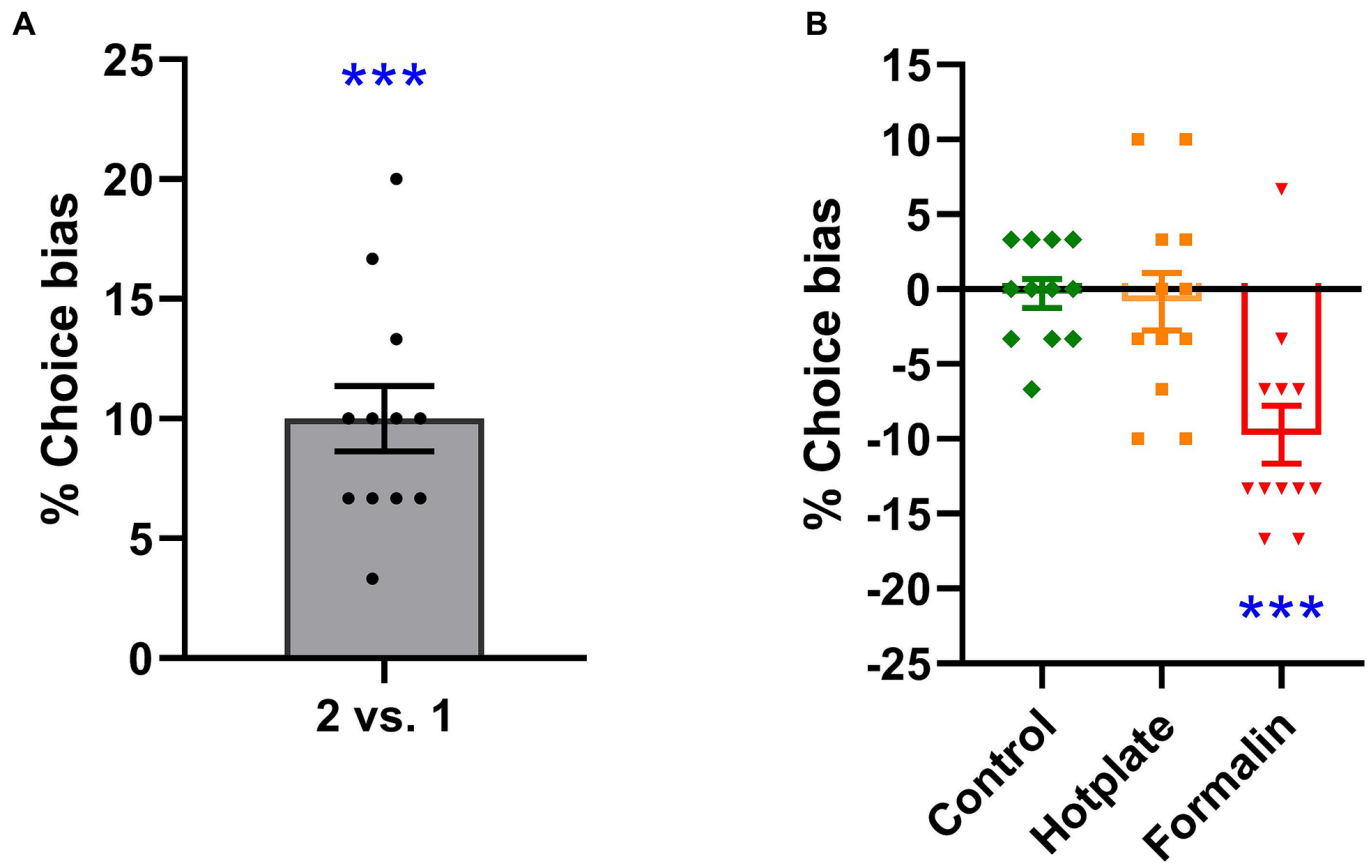
Effects of reward value and affective state manipulation on choice bias. Increasing the absolute reward value from one to two pellets resulted in a significant choice bias, which significantly differed from the 0% choice bias [one-sample *t*-test, *t*_11_ = 7.349, *p* < 0.001, *d* = 2.121, power = 1.000, **(A)**]. In the absence of any treatment, rats did not exhibit a significant choice bias [one-sample *t*-test, *t*_11_ = 0.291, *p* = 0.777, *d* = −0.084, power = 0.058, **(B)**]. Hotplate treatment did not induce a significant choice bias [one-sample *t*-test, 0.432, *p* = 0.674, *d* = −0.125, power = 0.068, **(B)**]. Formalin treatment resulted in a significant negative choice bias [one-sample *t*-test, *t*_11_ = 4.999, *p* < 0.001, *d* = −1.443, power = 0.995, **(B)**]. ***p < 0.001. The data is presented as the mean of % choice bias ± SEM (*n* = 12).

Rats exhibited a significant positive choice bias in favor of the substrate paired with two pellets over the substrate paired with one pellet (*t*_11_ = 7.349, *p* < 0.001, *d* = 2.121, power = 1.000, Figure 2A). A one-sample *t*-test against a theoretical mean of 0% revealed the significant effect of doubling the absolute value of the reward.

### Hotplate and formalin-induced acute pain in rats

**Figure 3:**
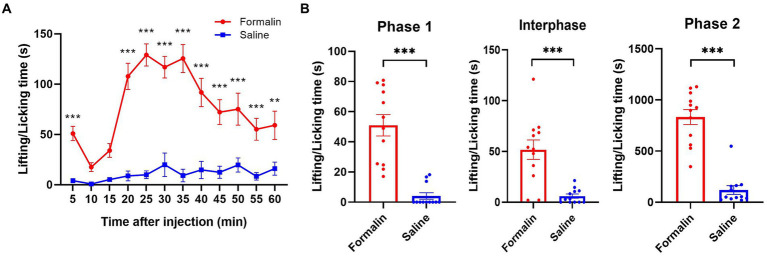
Nociceptive behaviors following the injections of formalin or saline. Rats were administered 1% formalin solution (50 μL) into one hind paw on the treatment day and an equal volume of saline (50 μL) on the same hind paw on the control day. Nociceptive behaviors were assessed by measuring the time spent on lifting and licking the injected paw. **(A)** The formalin injection resulted in significantly more nociceptive behaviors during the 60-min observation period compared to the saline injection. **(B)** The cumulative paw lifting and licking times across phases 1, interphase, and phase 2 after the formalin injection were significantly higher than those following the saline injection. *p < 0.05, **p < 0.01, ***p < 0.001. The data is presented as mean ± SEM (*n* = 12).

As for acute hotplate treatment, the rats displayed pain-related behaviors like licking or lifting their hind paws or jumping. The average latencies of rats to display pain-related behaviors on the hotplate were recorded in Supplementary Table S2. As for acute formalin treatment, it was observed that rats exhibited a typical two-phase pattern of nociceptive behavior following formalin injection into the hind paw. This response was significantly more pronounced than did rats injected with saline [treatment effect: *F*_(1, 11)_ = 74.853, Greenhouse–Geisser corrected *p* < 0.001, *η_p_^2^* = 0.773, power = 1.000; time effect: *F*_(11, 121)_ = 12.967, Greenhouse–Geisser corrected *p* < 0.001, *η_p_^2^* = 0.371, power = 1.000; interaction effect: *F*_(11, 121)_ = 9.452, Greenhouse–Geisser corrected *p* < 0.001, *η_p_^2^* = 0.301, power = 1.000; see Figure 3A]. In detail, a significant increase in the cumulative time spent on paw lifting and licking behaviors was observed across all phases following formalin injection compared to saline injection: specifically, phase 1 [0–5 min; paired *t*-test, *t*_(11)_ = 7.951, *p* < 0.001, *d* = 2.295, power = 1.000; Figure 3B, left panel], the interphase period [5–15 min; paired *t*-test, *t*_(11)_ = 5.141, *p* < 0.001, *d* = 1.484, power = 0.999; Figure 3B, middle panel], and phase 2 [15–60 min; paired *t*-test, *t*_(11)_ = 9.539, *p* < 0.001, *d* = 2.753, power = 1.000; Figure 3B, right panel].

### Pain-induced affective biases in rats

Acute hotplate-induced pain did not result in a significant choice bias in rats when choosing between the hotplate treatment-related substrate and the control treatment-related substrate (*t*_11_ = 0.432, *p* = 0.674, *d* = −0.125, power = 0.068, Figure 2B). In contrast, acute formalin-induced pain elicited a significant preference choice bias in rats, favoring the control treatment-related substrate over the formalin treatment-related substrate (*t*_11_ = 4.999, *p* < 0.001, *d* = −1.443, power = 0.995, Figure 2B).

### Trials to criterion, choice latency, and omissions

No significant effects on “trials to criterion” or “omissions” were observed following formalin treatment, while a significant prolonged effect of latencies was observed after formalin treatment (*t*_11_ = 2.845, *p* = 0.016, *d* = 0.821, power = 0.846). No significant effects on “choice latency,” “trials to criterion,” or “omissions” were observed following acute hotplate treatment or in 2 pellets vs. 1 pellet test, as summarized in Table 1.

**Table 1:** Results for trials to criterion, choice latency, and omissions in each test.

Test	Trials to criterion		Choice latency (s)		Omissions	
	Manipulation	Control	Manipulation	Control	Manipulation	Control
2 pellets vs. 1 pellet	6.38 ± 0.64	6.71 ± 1.14	2.17 ± 0.53	2.38 ± 0.47	0 ± 0	0 ± 0
Hotplate	6.63 ± 1.26	6.92 ± 1.02	1.81 ± 0.26	1.82 ± 0.34	0 ± 0	0 ± 0
Formalin	6.79 ± 1.34	6.38 ± 0.88	2.95 ± 2.04*	1.73 ± 0.70	0.33 ± 0.89	0 ± 0

The data represents the mean values derived from two pairing sessions conducted under each condition. Formalin treatment tended to increase the choice latencies in rats. No significant effects on trials to criterion, choice latencies, or omissions were observed in the hotplate test or the 2 pellets vs. 1 pellet test. The data is presented as the mean ± SEM (*n* = 12). Significance levels are indicated as ^*^p < 0.05, calculated using paired *t*-tests.

## Discussion

Our study found that acute formalin-induced pain in rats led to a discernible memory bias, as evidenced by a significant preference for the control treatment-related substrate over the formalin treatment-related substrate in the ABT. However, acute hotplate-induced pain did not produce a similar memory bias. This observation carries substantial implications for research in the emotional and cognitive aspects of pain. Previous studies have shown that cognitive bias testing, including ABT, can serve as a tool for assessing negative affective states caused by animal neuropsychiatric disorders (Zhang et al., 2022). Multiple studies by Robinson’s research group found that inducing animals to be in a putative negative affective state, such as through the administration of corticosterone, or exposure to acute psychosocial stress, elicits negative affective biases (Stuart et al., 2013; Hinchcliffe et al., 2017; Stuart et al., 2017). Conversely, inducing animals to experience putative positive affective states, such as through the administration of antidepressants like fluoxetine, sertraline, or duloxetine, or exposure to an enriched environment, results in positive affective biases (Stuart et al., 2013; Refsgaard et al., 2016; Hinchcliffe et al., 2022). Our study suggests that ABT can also be used to assess the negative affective states associated with acute pain, at least formalin-induced pain.

We did not detect significant memory biases induced by acute hotplate treatment that could be measured by ABT. The decision to employ thermal stimulation through the hotplate was based on established protocols and previous studies for using hotplate tests to check the animal’s sensitivity to heat pain (Lemberg et al., 2006). In the hotplate treatment days, rats are positioned on a heated surface, and when the rats display pain-related behaviors like licking or lifting their hind paws or jumping, they are promptly taken off the hotplate. As a result, the painful stimuli experienced by the rats in this test are escapable and of short duration (Bannon and Malmberg, 2007). The negative affective state induced by this acute pain stimulation may not continuously and enduringly affect the reward processing-related learning and memory stages in rats. Furthermore, it is important to consider that various types of pain stimuli activate distinct physiological and neural mechanisms, which may account for the observed disparity in our results. The hotplate treatment predominantly engages mechanical and thermal stimulation receptors, with their primary response occurring at the spinal cord level, initiating a sequence of early pain conduction events (Donnerer and Liebmann, 2013; Donnerer and Liebmann, 2018). In contrast, formalin, being an irritant compound, elicits pain perception upon injection and can lead to the phenomenon of central sensitization (Coderre and Yashpal, 1994; Vaccarino and Chorney, 1994). This central sensitization may influence higher-level brain functions, including learning and memory processes, resulting in memory bias.

To ensure the integrity of our study and to identify potential confounding factors, we took measures to detect non-specific effects caused by treatment, including attention distraction, changes in motivation, and alterations in motor ability. Previous research has reported that formalin injection in rats could lead to increased latency and omission of responses in behavioral tasks, indicating the presence of non-specific effects caused by pain (Boyette-Davis et al., 2008). In our experimental design, we employed several parameters to capture and assess the possible non-specific effects induced by the treatment. Specifically, we employed “trials to criterion,” “choice latency,” and “omissions” as indicators to assess these effects. Only formalin treatment led to a notable increase in choice latencies, whereas other indicators were not significantly influenced by any of the treatments. “trials to criterion” and “omissions” are reflective of the rats’ learning and memory abilities. Notably, there was no significant difference observed between the treatment group and the control group, suggesting that the treatment did not significantly impact the rats’ learning and memory capabilities. The observed increase in latencies of rats approaching the reward following formalin injection may be attributed to motivational conflict. We observed that rats exhibited pain-related behaviors, such as licking the injected foot to alleviate discomfort when conducting ABT. This is conflicted with their motivation to seek the reward, resulting in an extended latency to locate the reward. It should be noted that the rats in the formalin treatment condition also successfully found the reward within the prescribed time, supporting our overall conclusion that formalin induces a memory bias in rats based on the reward value.

It is important to use different tools to reflect the different dimensions of pain. Studying pain should not simply use reflex-based measurement methods, such as mechanical abnormal pain and thermal hyperalgesia (Li, 2013, 2015). As we mentioned earlier, pain not only includes sensory components, but also emotional and cognitive components. In human research, the negative affective states and cognitive changes caused by pain can be reflected through subjective scoring or self-report (Hjermstad et al., 2011), while in animals, studying the emotions and cognition of pain seems to be a challenging issue (Turner et al., 2019). Previous studies have tested the negative affective state caused by pain, such as the forced swimming test to reflect despair in animals under pain (Li and Chou, 2016), or the conditioned place avoidance test to reflect pain aversion and avoidance (De Felice et al., 2013). We used the ABT to measure the affective state of the rats under pain, and we focused on memory bias. The affective state of the rats during the learning stage would affect the experience of learning the rewarding paired substrate, and then affect the choice of the rats when they meet the previously rewarding-paired substrate. Taking into account that the emotional and cognitive dimensions of pain may require richer tools to measure, the use of ABT will be an important supplement in future animal pain research.

The investigation of memory bias in pain is of paramount significance. Empirical evidence from human studies has indicated that pain can induce a memory bias, thereby leading to a difference between actual experiences and memories (Berger et al., 2018). For instance, research has shown that individuals with somatoform pain disorder recalled fewer positive words in the free recall task (Pauli and Alpers, 2002). Another study discovered that individuals with chronic pain retrospectively rated the emotional valence of life events as more negative when experiencing pain compared to periods without pain (Meyer et al., 2015). On the other hand, memory bias can also affect pain. Memory biases in pain may exacerbate or sustain the pain experience (Schoth et al., 2020). The modification of memory biases could potentially serve as a valuable method in pain management (Siqveland et al., 2019). Consequently, a comprehensive understanding of the intricate interplay between pain and memory bias holds the potential for enhanced pain management strategies and improved therapeutic outcomes (Mazza et al., 2018).

Our study has limitations. Firstly, we only induced acute pain with hotplate or formalin and did not use other forms such as mechanical or electrical due to apparatus limits. The intensity of these stimuli could demand repeated intertrial stimulations, possibly disrupting ABT training. Future studies call for a specially adapted chamber to enable diversified pain modalities on memory bias exploration. Secondly, we did not incorporate any forms of treatments to improve the affective state of the rats subjected to acute pain, which might limit our understanding of the prevention and management of pain-induced memory biases. Lastly, our study focused solely on healthy subjects for baseline memory biases, further research should include subjects with conditions. For example, studying memory biases in animals with chronic pain after acute treatment with analgesics or antidepressants can help us understand the complex interplay between pain and emotion.

In conclusion, our study demonstrated that acute formalin-induced pain in rats led to a discernible memory bias, while acute hotplate-induced pain did not produce a similar memory bias. These findings will broaden our comprehension of the emotional and cognitive aspects involved in acute pain.

## Data availability statement

The raw data supporting the conclusions of this article will be made available by the authors, without undue reservation.

## Ethics statement

The animal study was approved by the Institutional Animal Care and Use Committee of the Chinese Academy of Sciences. The study was conducted in accordance with the local legislation and institutional requirements.

## Author contributions

YHZ: Methodology, Writing – original draft, Writing – review & editing, Data curation, Formal analysis, Visualization. JXL: Data curation, Formal analysis, Methodology, Writing – review & editing. NW: Conceptualization, Funding acquisition, Resources, Supervision, Writing – review & editing. JYW: Conceptualization, Funding acquisition, Resources, Writing – review & editing. FL: Conceptualization, Funding acquisition, Resources, Writing – review & editing.
