# Supplementary material for: Assessing cognitive biases induced by acute formalin or hotplate treatment: an animal study using affective bias test
Source: Front Behav Neurosci. 2024 Jan 25;18:1332760. doi: 10.3389/fnbeh.2024.1332760 (PMC10850345; doi:10.3389/fnbeh.2024.1332760)
Supplement: Supplementary file 1 [file Table_1.docx]

**Supplementary Table S1: Details of the substrates used in the experiment**

|  | Substrate A | Substrate B | Substrate C (blank) |
| --- | --- | --- | --- |
| Test 1: 2 pellets vs.1 pellet | Paper scraps | Cottonwood fiber | Timothy grass |
| Test 2: Control vs. Control | Aspen wood chips | Surgical mask scraps | Cotton balls |
| Test 3: Hotplate vs. Control treatment | Shredded apple wood chips | Plastic foam scraps | Glove scraps |
| Test 4: Formalin vs. Saline | Small oak blocks | Cardboard scraps | Pinewood grains |

The table lists the specific names of the substrates used in each test.

**Supplementary Table S2: Average latencies of each rat on the hotplate**

| Subject | Treatment: Hotplate of 52.5°C |
| --- | --- |
| 1 | 12.23 |
| 2 | 8.32 |
| 3 | 7.97 |
| 4 | 9.96 |
| 5 | 12.18 |
| 6 | 6.44 |
| 7 | 8.00 |
| 8 | 9.52 |
| 9 | 10.42 |
| 10 | 11.06 |
| 11 | 7.45 |
| 12 | 9.83 |

The table presents the average latencies of each rat on the hotplate. The average latencies are calculated as the mean of the first and second latencies recorded for each rat on the hotplate.
